# Current Status and Future Perspectives of Wearable Technologies for Oral Health in Clinical Applications

**DOI:** 10.3390/diagnostics16071015

**Published:** 2026-03-27

**Authors:** Yao Li, Mu Wang, Siqi Qiu, Jinyan Chen, Feng Wang

**Affiliations:** 1 School of Stomatology, Nanjing Medical University, Nanjing 210029, China; 23080302@stu.njmu.edu.cn; 2 Department of Stomatology, Peking Union Medical College Hospital, Chinese Academy of Medical Sciences, Peking Union Medical College, Beijing 100006, China; wangmu@pumch.cn; 3 Oral and Maxillofacial Surgery, Faculty of Dentistry, The University of Hong Kong, Hong Kong SAR, China; qiu-siqi02@connect.hku.hk (S.Q.); cccccccjy@connect.hku.hk (J.C.)

**Keywords:** oral wearable devices, human clinical trials, clinical applications, disease diagnosis, health management

## Abstract

This review aims to assess the clinical performance and application results of oral wearable devices in in vivo trials. Following a systematic search of PubMed, Cochrane, Embase, and Scopus databases up to 15 October 2025, and strict screening in accordance with PRISMA 2020 guidelines, 13 in vivo human trials were finally included for analysis. These were analyzed across four clinical functions: diagnosis, treatment, monitoring, and prevention. These devices have evolved from bulky prototypes into miniaturized, wireless systems with diverse diagnostic and therapeutic functions. Their applications now extend beyond common conditions like caries and bruxism to postoperative recovery and pediatric dental anxiety intervention. The findings show that some devices already offer practical value for clinical screening and auxiliary diagnosis. They demonstrate significant potential in early disease detection and medical cost control. However, development still faces many challenges. Technical issues include limited battery life, insufficient mechanical durability, and wireless transmission constraints within the oral environment. Furthermore, clinical evidence levels remain low, indications are narrow, and dedicated ethical and regulatory frameworks are lacking. Inconsistent regulatory standards, production costs, and clinician adoption hurdles slow its commercial development. In the future, the integration of AI, breakthroughs in energy harvesting, and the creation of digital health platforms will be key to overcoming technical bottlenecks.

## 1. Introduction

In recent years, the emergence of oral wearable devices has transformed traditional diagnostic and therapeutic paradigms of dentistry. In conventional dentistry, diagnosis relies heavily on clinical examinations (e.g., probing and visual inspection) and radiographic imaging (e.g., X-ray and CT). However, these “gold standards” are often limited by point-of-care sampling within the clinic, failing to capture the complexities of the dynamic oral microenvironment. Oral wearable devices are electronic systems that integrate micro-sensors to enable real-time, continuous monitoring and transmission of health data by being worn intraorally or on facial regions. They have helped clinical practice change from subjective assessment to objective metrics, from single-time examinations to continuous monitoring, and enabled integration with machine learning techniques [1].

Wearable devices have been increasingly applied in oral healthcare. Continuous monitoring of intraoral data may contribute to the prevention of oral diseases, such as dental caries [2] and oral inflammation [3]. At the same time, these technologies play a positive role in the diagnosis of periodontal disease [4], bruxism [5], and dental enamel demineralization [6], and can also support the treatment of orthodontic conditions [7], post-extraction pain [8], and periodontal diseases [4]. According to a recent systematic review [9], the potential of artificial intelligence (AI) in the dental field is being rapidly recognized. The integration with AI is expected to be a key direction for the evolution of oral wearable devices. These advances highlight the considerable potential of wearable devices to promote progress in healthcare. This review aims to evaluate the development and clinical applications of oral wearable devices, and to explore the factors limiting their progress, helping dental practitioners understand the current status of the field and providing guidance for future research. It hypothesizes that intraoral wearables can provide significant auxiliary value and potentially replace conventional protocols in specific clinical scenarios. It will first briefly review the developmental history of oral wearable devices and then focus on studies that have entered in vivo trials and analyze both the benefits and limitations of these devices in oral diagnosis and treatment. Finally, the future prospects and potential evolutionary trends of oral wearable technology will be discussed.

## 2. Historical Development of Oral Wearable Devices

The concept of oral wearable devices did not originate in the 21st century alongside the widespread adoption of intelligent devices. Instead, its intellectual roots can be traced back to oral medical research conducted in the mid-20th century. Early research focused mainly on fundamental exploration and functional validation. Although devices were bulky and limited to single functions, they laid important theoretical and practical foundations for later technological development.

Dynamic changes in the intraoral environment—such as pH, temperature, and ion concentration—play a crucial role in maintaining oral health. As early as the 1950s, researchers began attempting to measure intraoral pH using intraoral sensors [10]. In the 1960s, Graf and Mühlemann were among the first to propose the concept of true intraoral salivary sensors [11,12]. They developed miniature electrodes that could be mounted on dental prostheses or orthodontic appliances and employed telemetry techniques to wirelessly transmit data, successfully enabling continuous monitoring of dental plaque pH and fluoride ion activity. This work is widely regarded as the origin of oral wearable devices [13]. However, research during this period was largely confined to in vitro experiments and technological development. The wearable devices were bulky, required external equipment support, exhibited limited battery life, and offered poor comfort, which collectively hindered their widespread adoption and commercialization [13].

Entering the twenty-first century, rapid advances in Micro-Electro-Mechanical Systems, wireless communication technologies (such as Bluetooth), and materials science propelled oral wearable devices into a new phase of technological integration and prototype innovation. The devices began to shift toward miniaturization, multifunctionality, and wireless operation. Numerous innovative prototypes emerged in the academic community.

The development of new technologies has driven the rapid advancement of this field. With improvements in battery technology and wireless communication, an ear-worn wearable device capable of measuring occlusal force has also been developed [14].

The 2012 study by Mannoor et al. is an example of the contribution of advanced materials to this field. In this study, graphene nano-sensors were successfully printed onto a water-soluble silk substrate and seamlessly “transferred” onto the tooth surface, enabling wireless and passive detection of oral bacteria [15]. This study was the first to show the possibility of directly integrating advanced nanomaterials onto tooth enamel for biosensing applications. It established an example for a “non-invasive, wireless, and in situ” paradigm for bioassays in the domain of oral wearable devices.

Although many innovative device concepts have been reported, most studies remain at the in vitro stage. To assess the clinical performance and application results of oral wearable devices in in vivo trials, this review conducted a literature search to identify and analyze studies that have reached the human clinical trial stage.

## 3. Methodology

Based on the preceding review of the technological evolution of oral wearable devices, this section aims to define and identify evidence with clinical reference value through a systematic search methodology. This study focuses specifically on devices that have transcended the laboratory stage and entered human clinical trials to evaluate their clinical efficacy.

A literature search was conducted in PubMed, Cochrane, Embase (Ovid), and Scopus databases up to 15 October 2025. The search terms covered wearable electronic devices and stomatology/oral health-related keywords. The following search string was utilized in PubMed: (“Wearable Electronic Devices”[Mesh] OR (wearable*[tiab] AND (device*[tiab] OR sensor*[tiab] OR technolog*[tiab] OR electronic*[tiab])) OR “fitness tracker*”[tiab] OR “activity monitor*”[tiab] OR smartwatch*[tiab]) AND (“Dentistry”[Mesh] OR “Oral Health”[Mesh] OR dentist*[tiab] OR dental[tiab] OR “oral medicine”[tiab] OR “oral health”[tiab] OR orthodont*[tiab] OR periodont*[tiab]).

Included studies were English-language in vivo trials focusing on oral wearable devices in human applications. Exclusion criteria applied to in vitro and animal studies, as well as secondary literature such as reviews, letters, and conference records. Additionally, non-English publications were omitted from this review. Furthermore, this review was supplemented by a manual screening of reference lists from relevant review articles.

**Figure 1:**
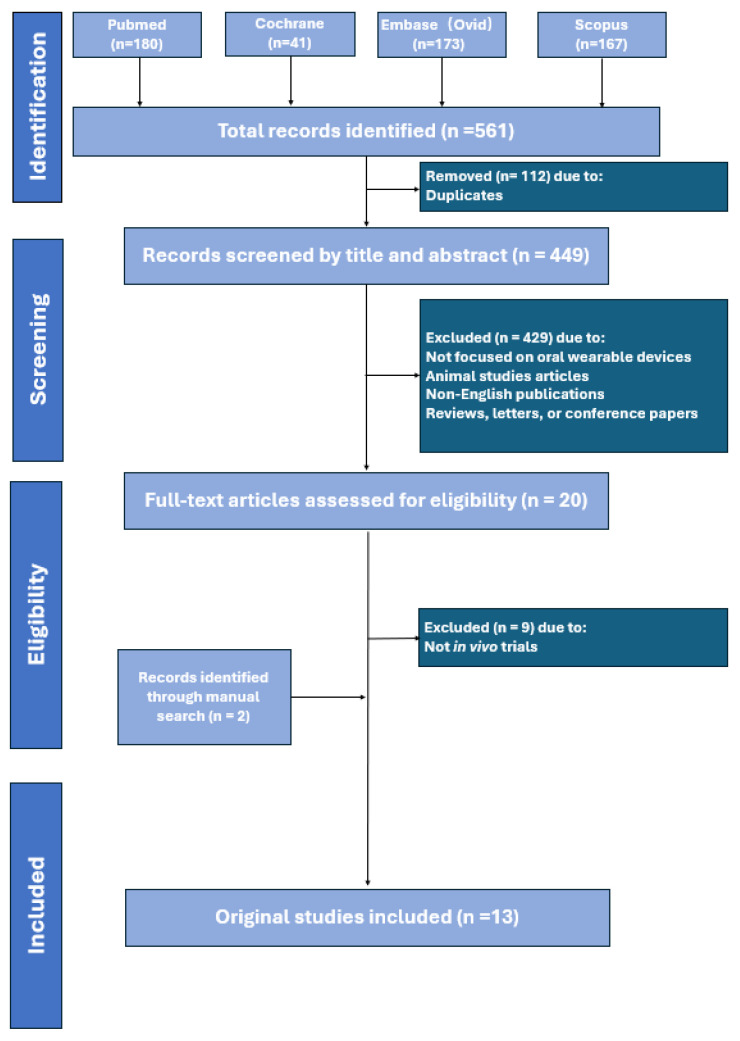
Flowchart of the study selection process.

After deduplication, 561 records remained from the initial pool across PubMed, Cochrane, Embase, and Scopus. Of the 561 unique records identified, 11 met the inclusion criteria, with 2 more added via manual search, totaling 13 included studies (Figure 1).

The study selection and reporting were conducted in strict accordance with the PRISMA 2020 guidelines. To ensure objectivity, the screening process was executed independently by two primary reviewers (YL and CJ). This involved an initial title and abstract screening, followed by a detailed full-text appraisal based on the predefined inclusion and exclusion criteria. Any discrepancies regarding study eligibility were resolved through discussion or, when necessary, by consulting a third senior reviewer to reach a final consensus.

The risk of bias and methodological quality of the 13 included papers were evaluated using RoB 2.0 and JBI critical appraisal tools (Table 1). The assessment covered multiple domains, including participant selection, measurement reliability, and statistical analysis. Overall, clinical validation studies demonstrated high quality, whereas early-stage technical validations exhibited a moderate risk of bias in terms of clinical evidence.

## 4. Results

### 4.1. Diagnostic Applications

Diagnostic application is one of the major research hotspots in the field of oral wearable devices. Many included studies aim to achieve early disease detection by monitoring chemical components. The oral chemical microenvironment strongly influences oral health, as distinct intraoral microenvironments provide favorable conditions for bacterial growth [26]. Alterations in the oral environment can disrupt the balance of the oral microbiota, leading to the overgrowth of potential pathogens and consequently increasing the risk of disease [27]. Precise monitoring of health indicators can be achieved through the detection of specific biomarkers, mediated by physical or chemical reactions triggered by functional materials. In 2020, a study reported a fluorescent mouthguard integrated with Zinc Oxide-Polydimethylsiloxane nanocomposites. This device enables the precise localization of dental lesions by monitoring the regional release of Volatile Sulfur Compounds (VSCs) [23]. Analogously, researchers have developed both composite-based mouthguards and facemasks capable of undergoing distinct colorimetric changes in response to Hydrogen Sulfide (H_2_S) gas [22,24]. This gas, a byproduct of microbial degradation at lesion sites, serves as a visual indicator for real-time diagnostic monitoring. While mouthguards provide direct intraoral sensing, functionalized facemasks offer a non-invasive alternative for monitoring exhaled H_2_S, reflecting the versatility of these responsive composites in various clinical scenarios. Despite their potential, these prototypes remain at the proof-of-concept stage (OCEBM Level 4). Their limited sample sizes necessitate large-scale validation before clinical implementation.

In addition to the early diagnosis of periodontal disease and dental caries, oral wearable devices have also been applied to the diagnosis of sleep-disordered breathing, particularly obstructive Sleep Apnea (OSA). A notable example is the work by Snow et al., who engineered a customized buccal oximeter integrated into an upper dental overlay. By providing continuous, multi-night Arterial Oxygen Saturation (SaO_2_) data, this reflective sensing platform offers a promising tool for the auxiliary diagnosis of OSA [19]. To validate the diagnostic accuracy of this device, a clinical trial was conducted involving 12 healthy subjects. The participants underwent six 7 min progressive hypoxia stages in a sedentary, semi-recumbent position. During the final 3 min of each stage, arterial blood samples were collected for CO-oximetry analysis. Comparison between the wearable oximeter and the gold-standard measurements demonstrated good accuracy and compliance with clinical requirements. Parallelly, wrist-worn devices have also been developed for monitoring oxygen saturation and pulse rate during sleep [17]. In vivo trials (OCEBM Level 2b) have indicated that such wearable systems offer high sensitivity and specificity for the screening of OSA.

Given that the diagnosis of malocclusion is an exacting process involving intricate occlusal examinations, the clear aligner investigated by Feng et al. offers a possible solution. By embedding high-performance piezoelectric sensors into the occlusal surfaces via Flexible Printed Circuits, the team developed a fully integrated, flexible, and self-contained clear aligner characterized by high sensitivity, a wide detection threshold, and multi-directional sensing capabilities [21]. Of particular significance is the integration of machine learning in this study. By analyzing occlusal data from more than 1400 malocclusion models, the system achieved a classification accuracy of 95% for malocclusion types and was also capable of identifying deleterious oral habits such as lip biting, thumb sucking, and bruxism.

Wearable devices can also assist in the diagnosis of bruxism. Flores-Ramirez et al. developed a portable oral monitoring device known as the Bruxist Activity Monitor System (BAMS), which is specifically designed to objectively collect and analyze parameters such as the number, duration, and inter-event intervals of occlusal events recorded during sleep cycles in patients with bruxism [20]. The system consists of an oral appliance embedded with two piezoresistive force sensors—corresponding to the bilateral mandibular second molars—along with a communication module and a user interface. During clinical trials, five complete sleep cycles were successfully recorded over a seven-day period. Throughout the monitoring process, the system demonstrated stable structural integrity and functional performance, with no significant data loss observed. This device enables portable, continuous, and accurate monitoring of nocturnal bruxism.

### 4.2. Therapeutic Applications

Progress has also been made in the use of wearable devices for the treatment of oral-related conditions. Stocchero et al. evaluated the clinical efficacy of a wearable Pulsed Electromagnetic Field (PEMF) therapy device [8]. In a double-blind study (OCEBM Level 1b), 114 patients undergoing mandibular third molar extraction were randomly assigned to an experimental group, a placebo group, and a control group. Under conditions in which all patients received antibiotics and analgesics, the incidence of wound dehiscence was significantly lower in the experimental group that wore the PEMF device for seven days, demonstrating its therapeutic effectiveness.

Another therapeutic-related study utilizes (OCEBM Level 1b) Virtual Reality (VR) to alleviate pediatric dental anxiety [16]. A study has shown that the use of VR displays is associated with reductions in physiological stress indicators in children, such as heart rate and perspiration. Compared to conventional treatment modalities, children utilizing VR interventions exhibited heightened levels of relaxation and tranquility throughout the perioperative period.

### 4.3. Monitoring Applications

These devices are specifically engineered to monitor intraoral data. For instance, an earphone-type sensor developed by Kurosawa et al. offers a distinct biomimetic approach for the indirect quantification of occlusal force [14]. It capitalizes on the dynamic morphological alterations of the external auditory canal during mandibular elevation to estimate occlusal loading. In vivo experiments involving five participants, each completing six trials, demonstrated a clear correlation between ear canal motion and occlusal force. This innovative device provides a novel, non-invasive solution for occlusal force measurement. Additionally, a miniaturized mouthguard-type sensing device was developed to continuously measure lingual pressure exerted on the palatal surfaces of maxillary anterior teeth during deglutition cycles [25].

Wearable devices are also capable of monitoring dietary behaviors and eating activities. A wrist-worn sensor employs a temporal convolutional network integrated with a multi-head attention mechanism to detect chewing actions, classify eating periods, and calculate eating speed [18]. Validation using 513 h of data collected from 61 participants demonstrated the effectiveness (OCEBM Level 2b) of this device in free-living conditions.

### 4.4. Preventive Applications

Currently, one approach for the prevention of oral diseases involves the use of wrist-worn platforms to capture hand kinematic data associated with oral behaviors, such as toothbrushing and dietary intake, thereby facilitating remote monitoring and targeted interventions for oral health. A representative example is a human activity recognition system that decodes inertial signals from smartwatches using machine learning algorithms to monitor toothbrushing compliance and foster the cultivation of optimal oral hygiene habits [2].

To provide a visual overview of the 13 included studies, this review summarizes them in Table 2, categorized by their clinical function.

To systematically evaluate the current state of oral-related wearable technology, the included studies were categorized based on their study design, functional purpose, and clinical readiness. Following the Oxford Centre for Evidence-Based Medicine (OCEBM) grading system, a hierarchy of evidence to distinguish between validated clinical applications and experimental research was established (Table 3).

## 5. Discussion

### 5.1. Clinical Significance of Oral Wearable Devices

Most studies that have entered in vivo trials to date have primarily focused on disease diagnosis. Currently, devices targeting OSA have demonstrated a high level of evidence. The cheek-mounted oximeter designed by Snow et al. [19] and the wrist-worn device designed by Jabbaripour et al. [17] have both been validated for efficacy through in vivo experiments (OCEBM Level 2b). This customized buccal oximeter overcomes several limitations of conventional pulse oximeters, as the buccal mucosa is richly vascularized, allowing for controlled temperature conditions, and will not be affected by ambient light. These validated devices offer reliable alternatives to traditional polysomnography. This implies that for patients suspected of having OSA, clinicians may no longer rely solely on expensive and inconvenient polysomnography in the future. Instead, preliminary screening and home monitoring could be conducted through these types of wearable devices.

BAMS [20] introduces a novel diagnostic approach for bruxism that diverges from traditional methods. Patients with bruxism often sleep alone and lack conscious awareness of their symptoms, resulting in low accuracy of self-reported assessments [28]. Consequently, clinicians primarily rely on medical history, physical examination, and objective monitoring methods for diagnosis [29]. The continuous monitoring of multiple occlusal events by BAMS makes it possible to shift the diagnosis of bruxism from subjective patient self-reporting toward objective clinical monitoring. The trial (OCEBM Level 2b) shows this can be a potential tool for the diagnosis of bruxism and for assessing its detrimental effects on dental structures, periodontal tissues, masticatory muscles, and temporomandibular joints. Moreover, its ability to operate continuously and reliably within the intraoral environment during overnight use represents a significant milestone in the adaptation of oral wearable devices to the challenging intraoral microenvironment.

Devices targeting dental caries [22,23,24], periodontal disease [24] and malocclusion [21] have also demonstrated their technical feasibility by in vivo trials(OCEBM Level 4). The mouthguards [23,24] provide direct intraoral sensing, functionalized facemasks [22] offer a non-invasive alternative for monitoring exhaled H_2_S, reflecting the versatility of these responsive composites in various clinical scenarios. The clear aligner was investigated by Feng et al. [21] provides a novel reference for the auxiliary diagnosis of malocclusion by incorporating machine learning algorithms. However, these prototypes remain at the proof-of-concept stage (OCEBM Level 4). Their limited sample sizes necessitate large-scale validation before clinical implementation.

In contrast to the extensive research dedicated to diagnostic modalities, in vivo trials focusing on the therapeutic phase of oral healthcare remain relatively sparse. To date, therapeutic investigations that have entered in vivo trials include wearable PEMF devices [8] and VR systems designed to alleviate anxiety in pediatric patients [16]. Validated by RCTs with a high level of evidence (OCEBM Level 1b), these two studies provide innovative treatment modalities for recovering from oral surgery and managing dental anxiety in children.

Included studies of monitoring applications are capable of continuous tracking of occlusal force [14], tongue pressure [25], and dietary behavior [18]. The former two studies represent exploratory research (OCEBM Level 4) focused on demonstrating the technical feasibility of real-time parameter quantification. These studies lay the technical foundation for potential clinical applications, such as the etiology of malocclusion, though their specific clinical implementation still requires further experimental development. In contrast, the latter not only exerts a positive influence on oral health management but also serves as a valuable monitoring tool for nutritional research and general health monitoring.

The wrist-worn device developed by González-Cañete et al. [2] prevents the occurrence of oral diseases and injuries resulting from improper brushing techniques by monitoring toothbrushing movements. However, this device remains in the experimental prototype stage (OCEBM Level 4), with research primarily concentrated on algorithmic validation.

Oral wearable devices enable the prevention and early detection of pathological changes. This not only improves patient prognosis but also yields significant economic benefits. While the average costs of late-stage restoration (dental implants) and mid-term intervention (root canal therapy) are approximately $2500–$4000 and $500–$1500, respectively, the cost of early prevention (routine cleaning) is merely $150–$300 [30]. The early monitoring advantages of oral wearable devices can effectively transform the need for restorative treatments costing thousands of dollars into low-cost preventive interventions.

A potential transformative impact of intraoral wearable devices on clinical practice lies in the integration of diagnosis and therapy into a single system. One typical example is a wearable hydrogel-based radiofrequency sensor that can adhere to tooth surfaces to enable in situ detection of H_2_S for the early diagnosis of periodontitis. Following wireless signal transmission to a mobile terminal, the device can trigger the release of antibacterial agents [4]. Although this system has demonstrated effectiveness in saliva sample testing, its clinical efficacy has yet to be validated. Similarly, a wearable multifunctional double-network hydrogel has been developed that combines visualized sensing of the acidic oral microenvironment with efficient photodynamic therapy [31], thereby enabling early detection and treatment of oral diseases. While these two studies delineate a promising trajectory for the evolution of intraoral wearables, their scope is currently confined to in vitro experimentation. This underscores a critical imperative for further robust investigation to bridge the gap between laboratory prototypes and clinical implementation.

### 5.2. Limitations and Challenges in the Development of Oral Wearable Devices

Oral wearable devices are required to operate within an exceptionally harsh and dynamic environment, which imposes significant challenges on their physical design and engineering performance.

Battery life limitations are a frequently mentioned concern. The intraoral environment imposes stringent constraints on device size and form factors, resulting in extremely limited space for battery integration. The battery capacity of intraoral devices is typically limited to approximately 40 mAh, which is substantially lower than that of conventional wearables [32]. This stringent constraint necessitates ultra-low power consumption to ensure an acceptable operational lifetime. However, many intraoral functions, such as neural stimulation, are inherently energy-intensive [33]. At present, some intraoral devices are powered by watch-type batteries [34]; however, their operational lifespan remains limited. Future research should focus on developing ultra-low-power operational modalities to address this challenge.

The sustained functional longevity of devices within the oral cavity requires them to endure chronic cyclical mechanical stresses from mastication, phonation, and bruxism, as well as the degradative potential of high humidity and acidic microenvironments. It is imperative for in vitro research to specifically evaluate the long-term reliability and performance stability of these devices under simulated intraoral conditions [35]. The intraoral environment is filled with saliva, muscles, soft tissues, and bone, all of which can cause significant attenuation of radiofrequency signals [36]. Signal loss during transmission through these biological tissues substantially affects communication range and stability [37]. Overcoming this limitation is essential for achieving real-time data transmission in intraoral wearable devices. The complex anatomical structure of the oral cavity constrains the development of intraoral imaging modalities, such as intraoral ultrasound systems. Achieving high-fidelity visualization of intricate intraoral anatomical structures necessitates devices with superior spatial resolution. However, the pronounced surface curvature of the oral mucosa, coupled with constant dynamic movement, poses a formidable challenge for real-time, high-precision intraoral imaging [38]. While conducting technical research and development, various experiments must also focus on the wearability and comfort of devices. Poor comfort can lead to suboptimal compliance [39]. Ultimately, the factor limiting therapeutic efficacy may not be technical limitations, but rather patient adherence.

Current clinical evidence remains largely at the feasibility-validation stage, characterized by a lack of multi-center, large-scale RCTs, which represent a critical entry point for future research. In addition, some trials are limited to short-term evaluations without long-term follow-up, resulting in insufficient evidence to support widespread clinical adoption. Also, most clinical trials focus on malocclusion, bruxism, dental care, and periodontal disease. This concentration is largely attributable to the fact that these conditions align well with the non-invasive, continuous, and accurate monitoring characteristics of oral wearable devices. Targeted investigations into other oral diseases remain scarce, indicating that substantial research gaps and opportunities still exist in this field.

Ethical considerations also matter. The rapid development of oral wearable devices has also given rise to a range of ethical concerns, including issues related to informed consent, data privacy, and algorithmic bias [40]. Informed consent is often undermined in wearable technology by a ‘click-to-agree’ formalism. Lengthy and obscure privacy policies fail to provide users with meaningful autonomy, rendering consent perfunctory rather than genuine [41]. Privacy concerns extend beyond data leakage to the unauthorized use of health data for non-medical purposes, including insurance pricing and employment screening [41]. The continuous collection of sensitive physiological data necessitates stringent encryption protocols and clear ownership frameworks to ensure patient autonomy and prevent unauthorized secondary use of personal health information. From a technical governance perspective, Differential Privacy [42] and Federated Learning [43] offer robust frameworks to safeguard sensitive information. By integrating noise or enabling decentralized training, these technologies address the inherent tensions between data utility and patient privacy. However, these two technologies have yet to achieve large-scale adoption on commercial platforms and continue to face a challenging trade-off between data utility and privacy preservation. Algorithmic bias represents a critical risk in AI-driven health analytics, potentially leading to significant disparities in diagnostic accuracy and treatment recommendations. Biases inherent in historical datasets may result in AI models that underrepresent diverse patient populations, thereby exacerbating existing health inequities [44].

The commercialization of oral wearables remains a major hurdle. From 2015 to 2024, despite numerous breakthrough device designations, only 12.3% achieved market access [45]. Divergent regulatory logic across jurisdictions increases global compliance costs and uncertainty.

In the US, the U.S. Food and Drug Administration (FDA) prioritizes algorithm validation and verification and clinical transparency for AI diagnostics [46], while making human factors safety assessments mandatory for home-use 510(k) premarket notification or De Novo reviews [47]. Since 2021, the EU’s Medical Device Regulation (2017/745) and the subsequent AI act have significantly raised requirements for clinical evidence, post-market surveillance, and Unique Device Identification, leading to more extensive and costly investigations [48]. Conversely, China’s National Medical Products Administration lacks a specific category for “oral wearable medical devices” [49], forcing firms to rely on analogical judgments based on intended use and invasiveness, which creates substantial regulatory uncertainty.

Production costs significantly constrain the development of oral wearables, with miniaturized, low-power sensors being the primary cost drivers. While the electronic components of wireless sensing systems benefit from economies of scale [50], the high-performance sensors themselves require specialized fabrication equipment that is heavily capital-intensive [51]. A major expense lies in reliable encapsulation to protect precision circuitry. Encapsulation, accounting for 80% of the total budget, is considered an industry standard [52], a figure that significantly exceeds the packaging overhead typically observed in standard consumer electronics. Furthermore, these devices frequently require semiconductor processes (e.g., photolithography, etching, thin-film deposition) or microfluidic fabrication. These methods demand massive capital investment and face significant challenges in yield control [51].

The commercialization of oral wearables as a standard of care hinges largely on dentists’ adoption. Surveys reveal that while 85% of dentists are familiar with teledentistry, fewer than 8% have received formal training [53]. Approximately 71.5% of practitioners express concern over the lack of in-person physical examinations, and 37% remain cautious or resistant toward integrating AI-driven decision support tools [54].

### 5.3. Future Perspectives on the Clinical Application of Oral Wearable Devices

AI and machine learning are expected to play a central role in the future development of oral wearable devices. AI- and machine learning-based algorithms are increasingly applied to process the complex, multidimensional data collected by these devices. For instance, AI can effectively dissect nuanced signals and capture complex patterns within salivary biomarkers, thereby enhancing diagnostic sensitivity and mitigating the confounding effects of inter-individual variability [55]. At present, AI has already been incorporated into experimental studies of oral wearable devices. Machine learning–based wrist-worn devices utilize inertial signal analysis to generate personalized notifications related to oral health management [56], such as reminders to replace toothbrushes. Similarly, pressure signals collected from wearable mouthguards have been combined with machine learning algorithms to classify types of malocclusions with high accuracy [21].

In addition to the devices currently discussed, research on oral health management using AI-driven or wearable technologies is also expanding. A recent study utilized Random Forest algorithms to analyze inertial signals from a combined smart toothbrush holder and wrist-worn sensor system, achieving a remarkable 96.13% accuracy in toothbrushing region recognition. This AI-driven approach can precisely identify 18 segmented dental areas while effectively filtering out ‘transition movements’, a common challenge in traditional monitoring due to data noise and coarse granularity [57].

A pivotal evolution in this field is the transition from passive “data monitoring” to active “behavioral modification” facilitated by AI-driven feedback loops. Current wearable technologies primarily focus on the objective recording of oral hygiene activities; however, their ultimate clinical utility lies in their ability to reshape user habits in real-time. Investigating real-time feedback mechanisms is poised to become a prominent trend in future research. For instance, devices could be designed to provide haptic alerts, such as vibrations, to immediately notify users when incorrect toothbrushing techniques are detected. In the future, wearable devices will evolve from passive diagnostic tools into active intervention systems capable of driving real-time behavioral modification.

To facilitate sustained intraoral performance, energy-harvesting technologies emerge as a promising strategy to circumvent the inherent constraints of battery capacity and achieve long-term power autonomy. Current research directions include harvesting mechanical energy generated by oral activities such as mastication and speech—via piezoelectric or triboelectric nanogenerators [58]—as well as exploiting intraoral temperature gradients using thermoelectric generators or chemical energy present in saliva through biofuel cells [59]. A recent study demonstrated a smart dental implant powered by human oral motion, which generated voltages ranging from 0.4 V to 1.3 V under simulated mastication, thereby validating the feasibility of this approach [60]. However, piezoelectric energy harvesting remains hindered by high impedance, regulation difficulties, and mechanical durability issues. Its high-voltage, low-current AC output necessitates complex power management, while long-term cyclic stress often leads to fatigue-driven material degradation. Similarly, thermoelectric harvesting is constrained by the narrow temperature gradients within the oral cavity; actual output is highly situational, relying on specific thermal fluctuations like drinking cold water [49]. Future research should focus on how to better integrate energy-harvesting technologies with oral wearable devices and validate their effectiveness. One study showcased a battery-free wearable dental patch for intraoral sensing and drug delivery [61]. The system employs near-field communication for wireless energy harvesting and data transmission, eliminating the need for bulky batteries, and integrates a low-power microcontroller unit to minimize power consumption. However, near field communication’s limited range (typically within a few centimeters) poses a significant bottleneck for the autonomous operation of oral devices. This is further compounded by electromagnetic absorption and scattering within the oral environment, which degrades power efficiency. These challenges remain to be addressed. Looking ahead, oral wearable devices are expected to function as data nodes within digital health platforms, providing patients with convenient health monitoring services. The integration and interoperability of oral health data with systemic health data could support comprehensive management of systemic diseases and facilitate a more holistic approach to healthcare [40].

### 5.4. Limitations of This Review

Several limitations of this review should be acknowledged. First, the literature search was restricted to English-language publications. The exclusion of non-English databases represents a potential “Tower of Babel” bias, particularly as East Asian countries like China and Japan are leading hubs for innovation in oral materials, MEMS technology, and AI-driven diagnostics. Consequently, a significant body of high-quality regional evidence may remain unaccounted for in this review.

The current evidence base is predominantly composed of small-scale, proof-of-concept trials (mostly OCEBM Level 4), with many studies involving very limited participants. This inherent lack of statistical power increases the risk of Type II errors and hinders the generalizability of the findings to broader patient populations.

Finally, given the rapid pace of technological iteration, a time-lag between trial completion and formal publication may exist, meaning some cutting-edge industrial developments might not be fully reflected in this review.

## 6. Conclusions

Oral wearable devices are transforming dentistry from subjective examinations to continuous, data-driven monitoring. Key findings indicate that research involving in vivo trials has demonstrated progress across diagnosis, treatment, monitoring, and prevention. While robust clinical evidence has been established in OSA monitoring and postoperative rehabilitation, evidence for early chemical diagnosis and precision prevention remains largely at OCEBM Level 4. These studies primarily focus on technical validation, and further clinical trials are essential to determine their definitive value in practice. Oral wearable devices are expected to become a core component of future diagnostic and therapeutic processes.

Despite these breakthroughs, the practical implementation of oral wearable devices faces severe challenges. The complex mechanical stress, high humidity, and acidic environment within the oral cavity impose stringent requirements on device durability. Furthermore, technical bottlenecks such as limited battery life, signal attenuation caused by biological tissues, and the difficulty of high-precision imaging of anatomical structures persist. At the industry level, issues such as restricted coverage of clinical indications, high production costs, and the lack of dedicated ethical and regulatory frameworks remain critical areas that future research must prioritize.

Future progress hinges on the integration of AI to enhance diagnostic precision, breakthroughs in energy harvesting, and the development of large-scale, more clinical trials to strengthen clinical evidence. As these technological and regulatory gaps are bridged, oral wearables are poised to become indispensable tools for both personalized oral healthcare and general health surveillance.

## Figures and Tables

**Table 1:** Methodological quality and risk of bias assessment of the included studies.

Reference	Study Design	Tool	Score	Grade
Stocchero et al. [8]	RCT	RoB_2.0	5/5	High
Trusculescu et al. [16]	RCT	RoB_2.0	4/5	Moderate
Jabbaripour et al. [17]	DTA	JBI	8/9	High
Wang et al. [18]	Diagnostic Cohort Study	JBI	8/9	High
Snow et al. [19]	Diagnostic Cohort Study	JBI	9/9	High
Flores-Ramirez et al. [20]	Diagnostic Cohort Study	JBI	6/10	Moderate
González-Cañete et al. [2]	Case Series	JBI	4/10	Low
Kurosawa et al. [14]	Case Series	JBI	6/10	Moderate
Feng et al. [21]	Case Series	JBI	7/10	Moderate
Zhang et al. [22]	Case Series	JBI	6/10	Moderate
Li et al. [23]	Case Series	JBI	5/10	Low
Ma et al. [24]	Case Series	JBI	5/10	Low
Matsumoto et al. [25]	Case Series	JBI	6/10	Moderate

**Table 2:** Summary of the included in vivo studies categorized by clinical functions.

Clinical Function	Reference	Clinical Application	Application Site	Key Parameters	Level (OCEBM)
Diagnostic Application	Li et al. [23]	Caries Localization	Teeth	VSCs	4
	Ma et al. [24]	Caries & Periodontal Disease	Teeth	H_2_S	4
	Zhang et al. [22]	Halitosis & Caries	Face (Integrated Mask)	H_2_S	4
	Snow et al. [19]	Sleep Apnea (OSA) Diagnosis	Buccal Mucosa	SaO_2_	2b
	Jabbaripour et al. [17]	Sleep Apnea (OSA) Diagnosis	Wrist (Wristband)	Pulse Oximetry and Heart Rate	2b
	Feng et al. [21]	Malocclusion Etiology	Dentition (Clear Aligner)	Occlusal Force, Malocclusion Classification	4
	Flores-Ramirez et al. [20]	Bruxism	Mandibular Molars (Intraoral Appliance)	Occlusal Events, Duration, Intervals	2b
Therapeutic Applications	Stocchero et al. [8]	Post-surgical Rehabilitation	Postoperative Site (Wearable Patch)	Wound Healing, Pain Reduction	1b
	Trusculescu et al. [16]	Pediatric Dental Anxiety	Head (VR Headset)	Stress Reduction	1b
Monitoring Applications	Kurosawa et al. [14]	Occlusal Force Monitoring	External Ear Canal (Earphone-type)	Dynamic Changes in Ear Canal Shape	4
	Matsumoto et al. [25]	Malocclusion Etiology	Lingual of Maxillary Incisors (Mouth Guard)	Tongue Pressure During Swallowing	4
	Wang et al. [18]	Dietary & Health Monitoring	Wrist (Wearable Motion Sensor)	Chewing Actions, Eating Speed, Duration	2b
Preventive Applications	González-Cañete et al. [2]	Oral Hygiene Management	Wrist (Smartwatch)	Brushing Compliance and Frequency	4

**Table 3:** Methodological characteristics, evidence levels, and clinical readiness of the included studies.

Reference	Study Design	Level (OCEBM)	Functional Category	Clinical Readiness	Subjects & Sample Size
Stocchero et al. [8]	RCT *	1b	Therapeutics	Validated Device	114 patients
Trusculescu et al. [16]	RCT	1b	Therapeutics	Validated Device	120 patients
Snow et al. [19]	Diagnostic Cohort Study	2b	Diagnosis	Validated Device	12 volunteers
Jabbaripour et al. [17]	DTA *	2b	Diagnosis	Validated Device	102 patients
Flores-Ramirez et al. [20]	Diagnostic Cohort Study	2b	Diagnosis	Validated Device	1 volunteer
Wang et al. [18]	Diagnostic Cohort Study	2b	Monitoring	Validated Device	61 volunteers
González-Cañete et al. [2]	Case Series	4	Prevention	Experimental Prototype	10 volunteers
Kurosawa et al. [14]	Case Series	4	Monitoring	Experimental Prototype	5 healthy subjects
Li et al. [23]	Case Series	4	Diagnosis	Experimental Prototype	8 volunteers
Ma et al. [24]	Case Series	4	Diagnosis	Experimental Prototype	9 volunteers
Zhang et al. [22].	Case Series	4	Diagnosis	Experimental Prototype	2 volunteers
Feng et al. [21]	Case Series	4	Diagnosis	Experimental Prototype	5 volunteers
Matsumoto et al. [25]	Case Series	4	Monitoring	Experimental Prototype	1 volunteer

* RCT: Randomized Controlled Trial; DTA: Diagnostic Test Accuracy Study.

## Data Availability

No new data were created or analyzed in this study. Data sharing is not applicable to this article.

## Abbreviations

The following abbreviations are used in this manuscript:

AI	Artificial Intelligence
BAMS	Bruxist Activity Monitor System
DTA	Diagnostic Test Accuracy Study
H_2_S	Hydrogen Sulfide
MMP-8	Matrix Metalloproteinase-8
OCEBM	Oxford Centre for Evidence-Based Medicine
PEMF	Pulsed Electromagnetic Field
OSA	Sleep Apnea
RCT	Randomized Controlled Trial
SaO_2_	Arterial Oxygen Saturation
VSCs	Volatile Sulfur Compounds
VR	Virtual Reality
